# Graph-based prediction of Protein-protein interactions with attributed signed graph embedding

**DOI:** 10.1186/s12859-020-03646-8

**Published:** 2020-07-21

**Authors:** Fang Yang, Kunjie Fan, Dandan Song, Huakang Lin

**Affiliations:** 1 School of Computer Science and Technology, Beijing Institute of Technology, 5 South Zhongguancun Street, Haidian District, Beijing, 100081 China; 2 Department of Biomedical Informatics, College of Medicine, The Ohio State University, Ohio, Columbus, 43210 USA

**Keywords:** Protein-protein interaction, Representation learning, Network embedding, Variational graph auto-encoder

## Abstract

**Background:**

Protein-protein interactions (PPIs) are central to many biological processes. Considering that the experimental methods for identifying PPIs are time-consuming and expensive, it is important to develop automated computational methods to better predict PPIs. Various machine learning methods have been proposed, including a deep learning technique which is sequence-based that has achieved promising results. However, it only focuses on sequence information while ignoring the structural information of PPI networks. Structural information of PPI networks such as their degree, position, and neighboring nodes in a graph has been proved to be informative in PPI prediction.

**Results:**

Facing the challenge of representing graph information, we introduce an improved graph representation learning method. Our model can study PPI prediction based on both sequence information and graph structure. Moreover, our study takes advantage of a representation learning model and employs a graph-based deep learning method for PPI prediction, which shows superiority over existing sequence-based methods. Statistically, Our method achieves state-of-the-art accuracy of 99.15% on Human protein reference database (HPRD) dataset and also obtains best results on Database of Interacting Protein (DIP) Human, *Drosophila*, Escherichia coli (*E. coli*), and Caenorhabditis elegans (*C. elegan*) datasets.

**Conclusion:**

Here, we introduce signed variational graph auto-encoder (S-VGAE), an improved graph representation learning method, to automatically learn to encode graph structure into low-dimensional embeddings. Experimental results demonstrate that our method outperforms other existing sequence-based methods on several datasets. We also prove the robustness of our model for very sparse networks and the generalization for a new dataset that consists of four datasets: HPRD, *E.coli*, *C.elegan*, and *Drosophila*.

## Background

Proteins are versatile macromolecules and perform a vast array of vital functions within organisms, and over 80% of proteins interact with other proteins while carrying out their functions [1]. Those interactions, known as protein-protein interactions (PPIs), are physical contacts of high specificity established between two or more protein molecules. PPI is of great importance in many cellular biological processes, including signal transduction, immune response, cell proliferation, DNA transcription, and replication. Analysis and elucidation of the PPIs provide valuable insights into the molecular mechanism and the protein functions [2]. In recent years, the rapid development of high-throughput technologies are used to detect protein interactions, such as yeast two-hybrid screens (Y2H) [3], tandem affinity purification (TAP) [4] and mass spectrometric protein complex identification (MS-PCI) [5], Tandem Affinity Purification and Mass Spectrometry (TAP-MS) [6], affinity chromatography and Co-Immunoprecipitation (Co-IP) [7]. These experimental methods have contributed to exponential growth of the number of PPIs of various species, but the functional annotation of both proteins and their interactions is updated at a slow speed. Meanwhile, these data suffer from problems including high false positives, false negative rate and low coverage [8]. To be more specific, although many protein-protein interaction links have been experimentally determined, the total number is still relatively few compared to the tremendous amount of links collected by the high-throughput technologies [9]. And these genome-scale experiments are costly, with inherent bias and limited coverage. The limitations of device resolution and environmental interference during operation will inevitably lead to errors and deviations in experimental techniques [10]. Therefore, high-throughput computational methods that are useful for the study of protein functions are required for discovering PPI with high quality and accuracy [11].

Recently, many high-throughput computational methods have been proposed. On the whole, they can be divided into two groups: classic machine learning algorithms and deep learning methods. For the first group, different machine learning methods were utilized for predicting PPIs to improve the efficiency and accuracy, such as decision trees [12], k-Nearest Neighbor (KNN) [13], naive bayes [14], random forest [15] and support vector machine (SVM) [16–18]. These features of these methods measure physicochemical properties of the 20 canonical amino acids, and aim at summarizing full sequence information relevant to PPIs. Compared to classic machine learning methods, deep learning methods are advantaged in extracting features directly from data and capture nonlinear dependencies between abstract features. They can also fully exploit the availability of the increasing large-scale and high-dimension raw datasets. Therefore, deep learning methods are unprecedentedly popular in recent years and have been successfully applied in various problems [19]. For PPI prediction, Sun et al. recently proposed a stacked auto-encoder (SAE) to study the sequence-based PPI prediction, which was the first to apply a deep learning algorithm to sequence-based PPI prediction and achieved promising results [20]. Du et al. proposed a method called Deep neural networks for Protein Protein Interactions prediction (DeepPPI), which employed deep learning to extract high-level discriminative features from common protein descriptors [21]. Lei et al. put forward a novel computational method based on Multimodal Deep Polynomial Network (MDPN) to encode multiple data from protein properties for PPIs prediction [22]. Hashemifar et al. presented a convolution-based model where feature extractions are terminated by processing data through an original randomly initialized and untrained matrix they named “random projection module" [23]. Next, a neural network based approach called Ensemble Deep Neural Networks (EnsDNN) was proposed to predict PPIs based on different representations of amino acid sequences [24]. Particularly, EnsDNN separately used auto covariance descriptor, local descriptor, and multi-scale continuous and discontinuous local descriptor, to represent and explore the pattern of interactions between sequentially distant and spatially close amino acid residues. Finally, Richoux et al. compared two carefully designed deep learning models and showed pitfalls to avoid while predicting PPIs through deep learning methods [25].

However, the deep learning algorithm presented by Sun et al. [20] and most of the methods we discussed above only considered sequence data, while the network data, such as their degree, position, and neighboring nodes in the graph, has been proved to be informative in PPI prediction. For example, *Licamele and Getoor* looked at the shared neighborhood among proteins and calculated the clustering coefficient among the neighborhoods for the first-order and second-order protein relations to predict the interactions in a yeast dataset [26]. Paradesi et al. identified nine structural features for *Saccharomyces cerevisiae* PPI networks and used them to learn classifiers for predicting new interactions [27]. You et al. developed a robust manifold embedding technique for assessing the reliability of interactions and predicting new interactions by utilizing the topological information of PPI networks [28].

The biggest challenge to apply graph-based deep learning methods for PPI prediction is the utilization way of the network information, that is, how to represent the graph structure of PPI network in low-dimensional embeddings, which should be used as feature inputs for downstream machine learning classifier. The good news is that there has been a surge in approaches that automatically learn to encode graph structure recently, using techniques based on deep learning and nonlinear dimensionality reduction. These methods are representation learning on graphs, which can be used to analyze social networks, molecular graph structures and recommender systems. The idea behind the representation learning approach is to learn a mapping that embeds nodes, or entire graphs, as points in a low-dimensional vector space. The purpose is to optimize the mapping so that geometric relationships in the learned space could reflect the structure of the original graph [29]. Representation learning has been successfully applied to link prediction, such as predicting missing friendship links in social networks [30] and inferring affinities between users and movies [31].

In this paper, by regarding PPI network as an undirected graph, we propose signed variational graph auto-encoder (S-VGAE), a representation learning model that could effectively take advantage of the graph structure and naturally incorporate protein sequence information as features. Our overall framework is composed of three parts. The first part is designed to code raw protein sequences, and the second part is the essential S-VGAE model used to further extract vector embedding for each protein with both graph structure and sequence information. The final part is a simple three-layer softmax classifier. Our S-VGAE model is designed based on the variational graph auto-encoder (VGAE) model proposed by Kipf and Welling [32], which is a framework that makes use of latent variables and is capable of learning interpretable representations for undirected graphs. To apply it to efficiently predict PPI, we primarily made three key improvements on the VGAE and greatly boosted the ultimate performance. Firstly, we modified the cost function to only consider those interactions with high confidence, which allowed us to learn accurate feature representation by focusing on high-confidence interaction information and was more robust to noise. In addition, we gave different signs to different interactions in the adjacency matrix so that the model could consider different impact of each interaction during the training process and strengthen the negative impact of the highly negative interactions. The last improvement was that we further train a neural network as the final classifier instead of using generative model to infer interactions. Since the input embedded representations already contained enough information, using the simple classifier is sufficient according to Occam’s Razor Principle [33].

## Results

Our work consists of three steps: basic protein sequence coding, graph-based feature extraction model, and the final neural network classifier. Firstly, we transform raw protein sequences into fixed-length coding using the conjoint triad (CT) method. Next, we propose an improved weighted variational graph auto-encoder (S-VGAE) to learn embeddings for each protein based on their sequence features and local graph information. Finally, we use these embeddings as inputs to train a simple feedforward neural network as the final classifier. In this section, we firstly evaluated the performance of the proposed method for predicting five different datasets: Human protein reference database (HPRD) dataset, Database of Interacting Protein (DIP) Human, *Drosophila*, Escherichia coli (*E. coli*), and Caenorhabditis elegans (*C. elegan*) by using different evaluation measures. We then compared the performance of the proposed method with existing methods from previous literature are presented. Finally, we discussed the robustness and the generalization of the proposed model.

### Evaluation criteria

In this paper, the performance of the proposed model was evaluated by means of the classification accuracy, specificity, sensitivity and precision, F-score value, as defined respectively by:

1 $$\begin{array}{*{20}l} {\kern5pt}Accuracy = \frac{TP + TN}{TP + TN + FP + FN} \end{array} $$

2 $$\begin{array}{*{20}l} Sensitivity = \frac{TP}{TP + FN} \end{array} $$

3 $$\begin{array}{*{20}l} Specificity = \frac{TN}{TN + FP} \end{array} $$

4 $$\begin{array}{*{20}l} {\kern3pt}Precision = \frac{TP}{TP + FP} \end{array} $$

5 $$\begin{array}{*{20}l} F-score = \frac{2 * Precision * Sensitivity}{Precision + Sensitivity} \end{array} $$

where TP, TN, FP and FN represent true positive, true negative, false positive, and false negative, respectively.

### Comparison with other methods

**Fig. 1:**
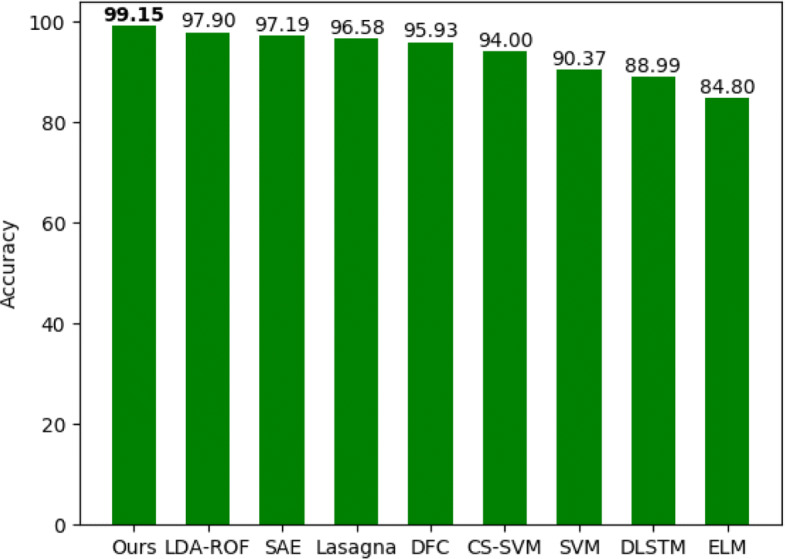
A detailed comparison of accuracy to several previous methods on the 2007 HPRD dataset

**Fig. 2:**
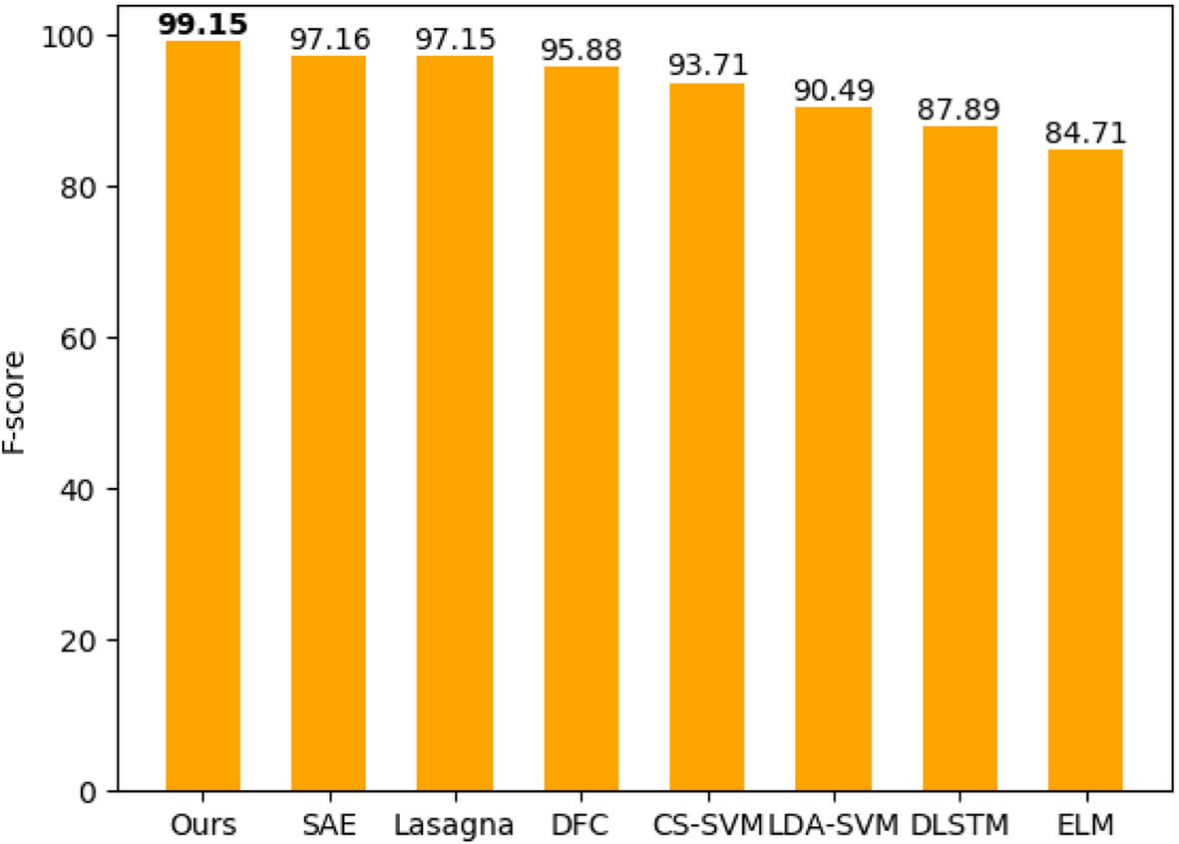
A detailed comparison of F1 score to several previous methods on the 2007 HPRD dataset

In order to demonstrate the performance of our model, we evaluated our model on five datasets as described in the “Datasets” section and compared our model to several popular methods. As indicated in Table 1, our method achieved above 98.5% accuracy on all datasets. For 2007 HPRD, *Drosophila* and *C.elegan* datasets, the F-score values of our model are more than 99%. As shown in Figs. 1 and 2, on the 2007 HPRD dataset, our model achieved state-of-the-art F-score value of 99.15% compared to eight popular existing methods. For example, Sun’s [20] obtained prediction F-score value of 97.16% and prediction accuracy of 97.19%. And Pan’s [34] work obtained prediction accuracy of 97.90% of latent dirichlet allocation-random forest (LDA-ROF) and prediction F-score value of 90.4% of latent dirichlet allocation-support vector machine (LDA-SVM) respectively. In summary, our model achieved the best prediction capacity.

**Table 1:** The performance of our model on five datasets

**Dataset**	Accuracy (%)	**Sensitivity (%)**	Specificity (%)	**Precision (%)**	**F1(%)**
HPRD	99.15 ±0.11	99.41 ±0.17	98.89 ±0.17	98.90 ±0.16	99.15 ±0.12
Human	98.79 ±0.07	98.00 ±0.21	99.58 ±0.17	99.57 ±0.17	98.78 ±0.24
*E.coli*	98.92 ±0.37	98.42 ±0.34	99.42 ±0.73	99.42 ±0.73	98.92 ±0.54
*Drosophila*	99.80 ±0.01	99.61 ±0.17	100 ±0.02	100 ±0.02	99.80 ±0.15
*C.elegan*	99.26 ±0.23	99.16 ±0.38	99.35 ±0.28	99.35 ±0.28	99.25 ±0.33

The detailed results of our method on the DIP Human, *E. coli*, *Drosophila* and *C. elegans* were listed in Table 2. Our model was compared against multiple baseline approaches, including: SAE [20], Lasagna [35], DeepPPI [25]. The results of SAE [20] were attained from the data provided by Guo et al. [18]. As the other methods of SVM [18], LDA-ROF [34], compressive sampling-support vector machine (CS-SVM) [36], and extreme learning machine (ELM) [37] were not conducted on these four datasets. Particularly, Richoux et al. [25] proposed to compare two different neural network architectures: Deep Fully Connected Network (DFC) and Deep Long Short Memory Network (DLSTM). Our model was compared with these two models respectively. In addition, since the DFC and DLSTM methods were applicable to large-scale datasets which were designed to solve the problem of information leak and didn’t avoid the underfitting in the small datasets, we didn’t use their results on *E. coli* and *C. elegans* datasets.

**Table 2:** The performance comparison of F1 score between our model and four existing sequence-based methods on four PPI datasets

**Method**	Human	***E.coli***	***C. elegan***	***Drosophila***
**Our model**	**98.78**	**98.92**	**99.25**	**99.80**
Lasagna [35]	97.25	89.92	98.40	98.89
DFC [25]	95.14	——	——	96.39
DLSTM [25]	89.10	——	——	91.05
SAE [20]	94.53	96.03	97.17	97.16

On the DIP Human dataset, our model yielded a F-score value of 98.78% which greatly outperformed the model named Lasagna [35] being 97.25% and the model named DFC [25] being 95.14%. For *E. coli*, our model achieved the F-score value of 98.92%, which was also significantly superior to other methods. For *Drosophila*, our model obtained the F-score value of 99.80% while for *C.elegans* the F-score value was 99.25%. It can be seen that our model has demonstrated promising results on several datasets and has proved its potential in PPI prediction regardless of the dataset’s size, coverage, and species.

### Robustness and generalization

First, we discuss the robustness of our model for very sparse datasets since the existing PPIs are limited [38]. We define *coverage* as the proportion of training samples to the number of total samples. The training set is selected as each protein includes *coverage* portion of its positive edges and highly negative edges. The less the *coverage*, the more sparse the training set.

**Fig. 3:**
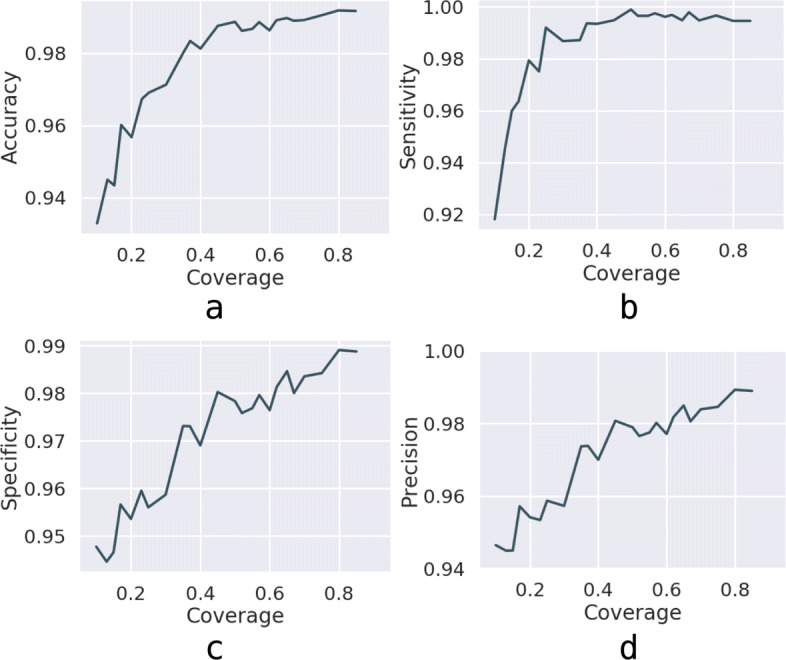
Relationship between four criteria and coverage. All the simulations were trained on the HPRD dataset for 50 iterations

As we can see from Fig. 3, the accuracy, sensitivity, specificity, and precision are all rising as the *coverage* increases. It can be observed from Fig. 3a that the accuracy is already above 93% even when the *coverage* is only 0.1, which illustrates that our model is effective even when the datasets are sparse and can be applied in real prediction of other species. Besides, as indicated in Fig. 3b, the sensitivity is up to 99% at the *coverage* of only 0.4. Figure 3c and d also demonstrate the robustness and validity of our model consistently.

To test the generalization, we combine HPRD, *E.coli*, *C.elegan* and *Drosophila* datasets into one larger dataset. This new dataset consists of four different species and contains 21881 proteins with 69550 positive samples and 69283 negative samples. We randomly split the dataset into 50% training samples and 50% test samples and this process is repeated five times. The training data was trained for 50 iterations and the average of five results denotes the final score. As we can see in Table 3, the accuracy, sensitivity, specificity and precision, F-score value are all more than 96%. Considering the heterogeneity and sparsity of this dataset, the performance is reasonable and desirable.

**Table 3:** The performance of our model on the combined new datasets

**Test set**	Accuracy (%)	**Sensitivity (%)**	Specificity (%)	**Precision (%)**	**F1 (%)**
1	97.61	97.91	97.31	97.33	97.62
2	96.60	97.88	95.31	95.44	96.64
3	97.07	97.58	96.55	96.60	97.09
4	96.78	97.17	96.40	96.44	96.80
5	97.73	97.84	97.61	97.63	97.73
Average	**97.16 ±0.50**	**97.68 ±0.31**	**96.64 ±0.90**	**96.69 ±0.86**	**97.18 ±0.41**

## Discussion

With a graph representation learning model, our method is demonstrated effective and robust in PPI prediction. However, the model still has great improvement space as follows. In our model, considering different impacts of different edges, we introduce the mechanism of confidence for each edge in the PPI network which can be reflected in the adjacency matrix ***A***. Currently, we divide all the edges into three groups and assign constant value for each edge in the same group. In the future work, the quality of each interaction should be taken into account and assigned a specific confidence value to make the prediction model more informative.

For protein sequence coding, we used the pre-defined CT method. Other popular coding methods such as auto covariance (AC) and local descriptor (LD), the manually constructed or selected representation is more or less polluted or biased, which would affect the learning ability of the deep learning method. But the CT method is based on rules and the error will be smaller than these two method. Therefore, developing more precise coding methods is crucial to further improve the model in our future work. As we all known, the sequence of nucleotides that forms a gene is first translated into an amino acid sequence, following the rules encoded in the genetic code. The corresponding linear chain of amino acids becomes functional only when it adopts a three-dimensional shape, the so-called tertiary, or native structure of the protein. These 3D structures of proteins provide the opportunity for in silico prediction methods. The opportunity is that if in silico methods can predict whether two given 3D structures interact, then these methods may be applied to predict interactions among the large amount of proteins with known or inferred 3D structure [39]. Our future work could integrate the text description information annotated in the database to the codings using natural language processing technique and the 3D structures of proteins to better represent the protein.

## Conclusions

In this paper, we proposed S-VGAE, an improved graph representation learning method, to incorporate graph information in PPI networks into PPI prediction. Then the abstract features are based on both sequence information and graph structure. Experimental results demonstrated that our method performed significantly well and outperformed other existing sequence-based methods on several datasets. We also proved the robustness of our model for very sparse networks and the generalization for different kinds of datasets. To the best of our knowledge, our method is one of the first models to apply graph-based representation learning technique, thus successfully apply a deep learning algorithm to graph-based PPI prediction. It is also anticipated that our method can be generalized to many other related bioinformatics studies. For example, we can conduct representation learning with graph and simplified molecular input line entry specification (SMILES) string features of drug molecules using S-VGAE to predict drug interactions. We can construct an undirected weighted graph, where each vertex represents one drug and each edge denotes one interaction between two drugs. After obtaining the SMILES strings of drug molecules as the input features of each node, we can apply S-VGAE to obtain the hidden representations of drugs and predict the interactions between them. Particularly, Drug-drug interactions (DDIs) are from DrugComb database [40] and DDIExtraction 2013 dataset [41] and molecular structures of drugs can be obtained freely from DrugBank.

## Methods

**Fig. 4:**
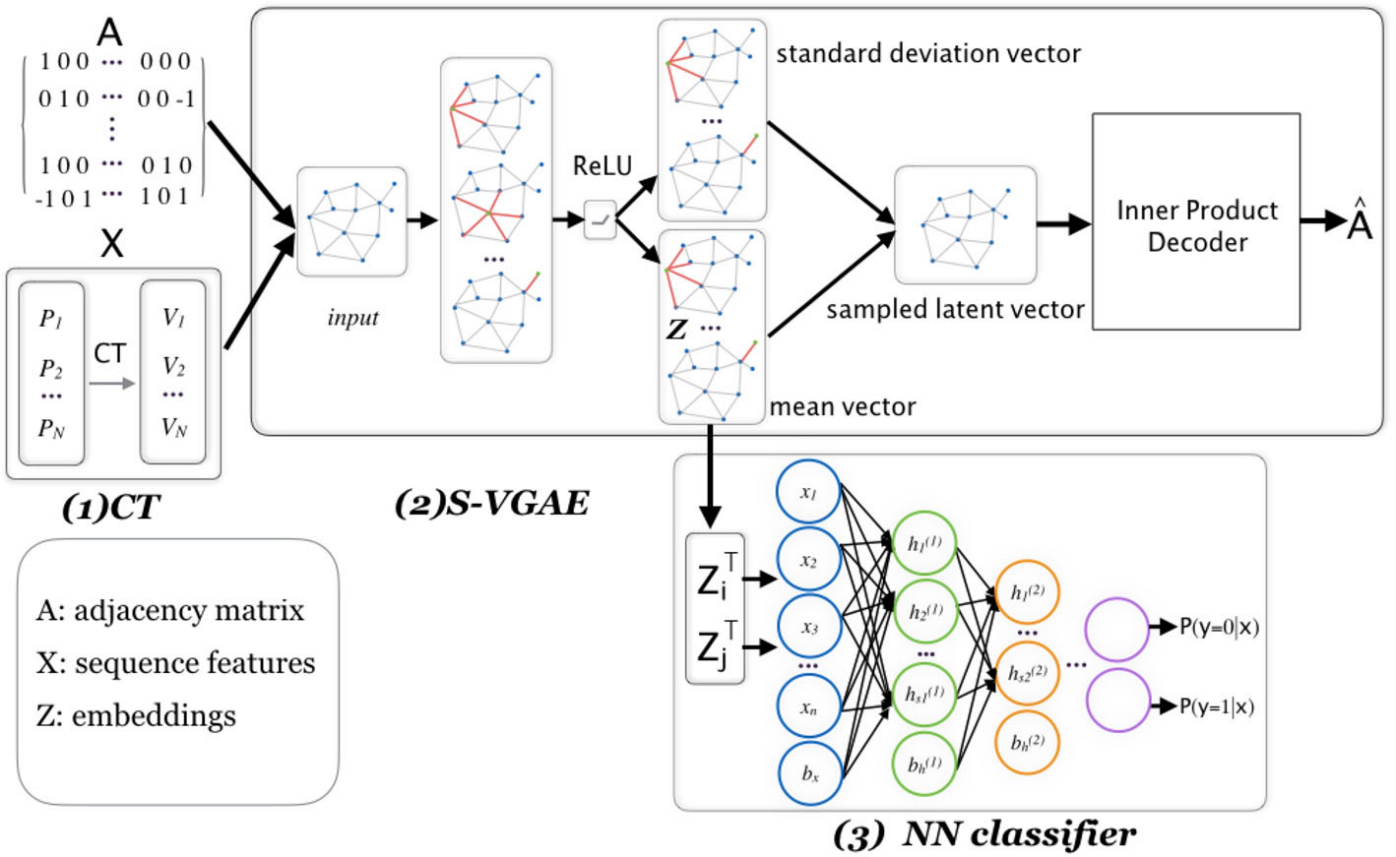
Our overall model architecture. The first part is CT used for protein coding. The second part is S-VGAE used for feature extraction. The final part is a simple neural network classifier used for prediction

Our overall framework consists of three steps as shown in Fig. 4: basic protein sequence coding, graph-based feature extraction model, and the final neural network classifier. The first step is to transform raw protein sequences into fixed-length codings in order for subsequent training. Next, we propose an improved weighted variational graph auto-encoder (S-VGAE) to learn embeddings for each protein based on their sequence features and local graph information, which is equivalent to the feature reduction and extraction. Finally, we use these embeddings as inputs to train a simple feedforward neural network as the final classifier. In this section, we also introduce the datasets and model settings.

### Datasets

#### Benchmark dataset

We used Pan’s [34] dataset from http://www.csbio.sjtu.edu.cn/bioinf/LR_PPI/Data.htmas the benchmark dataset. The positive samples in the dataset are from the human protein reference database (HPRD, 2007 version), with the elimination of the self-interactions and duplicate interactions. We finally obtained 36591 positive pairs. Based on the common assumption that two proteins in different cellular compartments do not interact, proteins used in constructing negative samples are selected by following the listed criteria: (1) Collecting human proteins annotated with “human” in the ID field only. (2) Excluding sequences annotated with ambiguous or uncertain subcellular localization terms, such as “potential”, “probable”, “probably”, “maybe”, or “by similarity". (3) Including those sequences marked with unique locations only. (4) Excluding sequences annotated with “fragments”, and eliminating sequences with less than 50 amino acid residues as they may only be fragments. (5) Proteins with unusual amino acids such as U and X were removed. The collected proteins were randomly paired with other proteins in different subcellular locations to generate negative samples. Finally, the total amount of the remaining negative samples were 36324.

#### Other datasets

We also used Guo’s [18] dataset to evaluate our model including: (1) Human dataset containing 9435 proteins with 37020 positive samples and 37027 negative samples. (2) *E. coli* dataset containing 1834 proteins with 6954 positive samples and 6954 negative samples. (3) *Drosophila* dataset containing 7059 proteins with 21975 positive samples and 21975 negative samples. (4) *C. elegan* dataset containing 2640 proteins with 4030 positive samples and 4030 negative samples. The negative samples used for training in each dataset were selected according to the same criteria presented in the above Benchmark Dataset part.

### Model settings

Our model was implemented using Tensorflow in Python and took advantage of the strong computing capacity of GPU. All the simulations were carried out on a computer with 4.00GHz 8-core CPU and 59GB memory. The GPU we used was NVIDIA GeForce GTX 1080 with 7GB memory. Our source code and datasets are available at https://github.com/fangyangbit/S-VGAE.

The S-VGAE model has two hidden layers with 96 neurons and 48 neurons of each layer respectively. The final softmax classifier has three hidden layers with 128, 64 and 32 neurons each layer and uses dropout technique during training in order to avoid overfitting. Dropout is a technique that randomly drops units (along with their connections) from the neural network during training [42]. For both parts, we initialize weights as described in [43] and train for 50 iterations using Adam algorithm with a learning rate of 0.005. We tuned the hyperparameters of our model to optimize system performance by conducting 5-fold sentence-level cross-validation on the training set. To determine the parameter, four-fifths of the whole dataset are randomly chosen to train the classifiers with different number of hidden nodes, while the rest one-fifths of the dataset are used as the validation set to compute the accuracy. For Adam optimization, we set the learning rate lr = 0.005 as suggested by Kingma et al. [44]. To alleviate the over-fitting problem, the dropout rate was set to 0.5 in our model, as used by Hinton et al. [45].

In our experiments, each dataset was randomly split into 80% training set and 20% test set. The model was trained and validated using 5-fold cross-validation, and the performance of our model was evaluated by the hold-out test set. In order to test the robustness of our method, this process of random selection was repeated five times. Therefore, five models were generated based on different training sets and the overall performance was the average of results on five different test sets.

### Protein sequence coding

There are several existing methods for protein sequence representation such as auto AC, CT, and LD [46]. In our model, we choose CT as our coding method. The CT method was first proposed by Shen et al. to code single protein [47]. The information of protein sequences can be projected into a homogeneous vector space by counting the frequencies of each triad type. The whole process is described as follows. First of all, all amino acids are clustered into seven categories according to their dipole and side chain volumes. The classification of amino acids is shown in Table 4 and the classification principle is described in detail in the paper [47]. Amino acids within the same group likely involve synonymous mutations due to their analogous characteristics. Then, each amino acid can be substituted by its category label and thus each protein is a string of integers. Next, a window of size three is used to slide across the sequence one step at a time and count the number of occurrences of each triad type. Since we regard any three continuous amino acids as a unit and the amino acids have been catalogued into seven classes, there are 7×7×7 different combinations, that is the size of CT vector is 343. The CT representation is defined as:

6 $$\begin{array}{@{}rcl@{}} V = [n_{0}, n_{1},\ldots, n_{q}] \end{array} $$

**Table 4:** Classification of Amino Acids according to their Dipoles and Volumes of the Side Chains

**Class**	**Amino Acids**
*C*_1_	Ala, Gly, Val
*C*_2_	Ile, Leu, Phe, Pro
*C*_3_	Tyr, Met, Thr, Ser
*C*_4_	His, Asn, Gln, Tpr
*C*_5_	Arg, Lys
*C*_6_	Asp, Glu
*C*_7_	Cys

where *n*_*i*_ is the number of occurrences of each triad type and *q* equals to 343.

### Our S-VGAE model

This is the core part of our overall framework. After initial coding of sequences by the CT method, we further conduct representation learning with graph and sequence features using our signed variational graph auto-encoder (S-VGAE) model. We will discuss this model by problem formulation, and then its inference part and generative part. The inference part is an encoder that encodes original proteins into embeddings while the generative part is a decoder that decodes the embeddings back into original proteins. The purpose of this model is to learn interpretable embedding for each protein by training encoder and decoder at the same time.

#### Problem formulation

We are given an undirected weighted graph $\mathcal {G} = (\mathcal {V}, \mathcal {E})$ with $N = | \mathcal {V} |$ nodes, thus *N* is the number of proteins, and each vertex of $\mathcal {G}$ represents one protein while each edge is one interaction. The adjacency matrix ***A*** of $\mathcal {G}$ is provided. In matrix ***A***,*A*_*ij*_ denotes whether there exists an interaction between protein *i* and protein *j*. We enforce self-loops in the graph by simply adding the identity matrix to ***A***. The input features of each node are included in an *N*×*R* matrix ***X***, which are sequence representations by the CT method and *R* equals to 343 in this case. The desired outputs of this model are latent variables ***z***_***i***_, summarized in an *N*×*P* matrix ***Z***, which will contain the embeddings of proteins we expect to get where *P* is the dimension of each embedding.

The model is basically an encoder-decoder approach. First the encoder maps each node *v*_*i*_ in the graph to a low-dimensional vector embedding, ***z***_***i***_, based on the node’s position in the graph, its local neighborhood structure, and its attributes. Next, the decoder extracts the classification label *A*_*ij*_ associated with *v*_*i*_ and *v*_*j*_ (i.e., the label of interaction between protein *i* and *j*). By jointly optimizing the encoder and decoder, the model learns to compress information about graph structure into the low-dimensional embedding space. The intuition behind this encoder-decoder idea is that if we can learn to decode hig-dimensional graph information from encoded low-dimensional embeddings, then, in principle, these learned low-dimensional vectors should contain all information necessary for downstream machine learning tasks, for example, classification.

#### The encoder

The inference module is a graph convolutional networks (GCNs) encoder [48], which is a function with the goal of a mapping from the original features ***X*** to embeddings ***Z*** with the augmented information of ***A***. In our current implementation, a simple model parameterized by a two-layer GCN is utilized:

7 $$\begin{array}{*{20}l} q(\boldsymbol{Z} | \boldsymbol{X}, \boldsymbol{A}) &= \prod_{i=1}^{N}q(\boldsymbol{z_{i}} | \boldsymbol{X}, \boldsymbol{A}) \end{array} $$

8 $$\begin{array}{*{20}l} q(\boldsymbol{z_{i}} | \boldsymbol{X}, \boldsymbol{A}) &= \mathcal{N}\left(\boldsymbol{z_{i}} | \boldsymbol{\mu_{i}}, diag\left(\boldsymbol{\sigma_{i}^{2}}\right)\right) \end{array} $$

where ***μ***=*GCN*_***μ***_(***X***,***A***) is the matrix of mean vectors ***μ***_***i***_ and log***σ***=*GCN*_***σ***_(***X***,***A***). The GCN model is defined as *GCN*(***X***,***A***)=***A****ReLU*(***AXW***_***0***_)***W***_***1***_, and ***W***_***i***_ are parameter matrices we need to train. In our model, *GCN*_***μ***_(***X***,***A***) and *GCN*_***σ***_(***X***,***A***) share ***W***_***0***_ in order to reduce parameters. *ReLU*(·)=*max*(0,·) is the activation function and $\mathcal {N}$ is the unit gaussian distribution. The intuition of GCN using *GCN*(***X***,***A***)=*ReLU*(***AXW***_***0***_) is as follows. The multiplication with adjacency matrix ***A*** means that, for every node, we sum up all the feature vectors of all neighboring nodes and itself. In this way, GCN can effectively learn embeddings through integrating neighboring graph features.

#### The decoder

The generative module we define here is a simple inner product decoder:

9 $$\begin{array}{*{20}l} p(\boldsymbol{A} | \boldsymbol{Z}) &= \prod_{i=1}^{N}\prod_{j=1}^{N}p(\boldsymbol{A_{ij}} | \boldsymbol{z_{i}}, \boldsymbol{z_{j}}) \end{array} $$

10 $$\begin{array}{*{20}l} p(\boldsymbol{A_{ij}} &= 1 | \boldsymbol{z_{i}}, \boldsymbol{z_{j}}) = \sigma(\boldsymbol{z_{i}}^{\top}\boldsymbol{z_{j}}) \end{array} $$

where *σ*(·) is the logistic sigmoid function. We use the inner product of two embeddings ***z***_***i***_ and ***z***_***j***_ as the probability of these two proteins existing the interaction. As indicated in Fig. 4, the output of the decoder $\boldsymbol {\hat {A}}$ is the approximation of adjacency matrix ***A*** and we optimize the model so as to make them as close as possible.

#### Training method and implementation details

In this section, we will detailedly discuss two improvements we proposed on VGAE and explain why they work in our application.

#### (1) cost function

As the whole interaction network is regarded as an undirected graph and each item in the adjacency matrix represents whether there exists an interaction between the two proteins, in our S-VGAE model, we define the cost function as:

11 $$\begin{array}{@{}rcl@{}} \begin{aligned} \mathcal{L} = \mathbb{E}_{q(\boldsymbol{Z} | \boldsymbol{X}, \boldsymbol{A})}\lbrack \log p(\boldsymbol{A^{\star}} | \boldsymbol{Z})\rbrack - KL\lbrack q(\boldsymbol{Z} | \boldsymbol{X}, \boldsymbol{A}) \| p(\boldsymbol{Z}) \rbrack \end{aligned} \end{array} $$

where *KL*[*q*(·)∥*p*(·)] is the Kullback-Leibler divergence between *q*(·) and *p*(·). The first term is to minimize the reconstruction error of the adjacency matrix ***A***. It should be noticed that, we only consider those interactions with high confidence, which we specify as ***A***^***⋆***^ (***A***^***⋆***^⊂***A***) to be reconstructed. The second term is to minimize the difference between *q*(***Z***|***X***,***A***) and *p*(***Z***). The cost function is the tradeoff between how accurate our model can be and how close the distribution of embeddings can match *p*(***Z***). In this case, we assume *p*(***Z***) as a Gaussian prior and the reparameterization trick is used for training [49].

As mentioned above, we only consider the cost of interactions with high confidence while ignoring those edges of uncertain confidence. In other words, we assume that those edges of uncertain confidence are random noises to our model and even disturb the training process thus affecting the ultimate performance. Therefore, we need to construct high-confidence sets from original datasets. For positive samples, the confidence are always high since they are actually observed. As for other items in the adjacency matrix ***A***, they are divided into two groups: the highly negative group and the uncertain group. The edges in the highly negative group are negative edges with high confidence, which are selected based on the criteria described in the “Datasets” section.

#### (2) signed adjacency matrix

During the training process, the adjacency matrix ***A*** plays an important role since it not only defines the cost function but also serves as a critical parameter in the GCN. The common adjacency matrix consists of only 0 and 1. However, as we discussed in the last section, different edges actually have different confidence and therefore should have different impacts on the learning process.

In order to embody the differentiated impacts, we assign positive edges positive values (1), the highly negative group negative values (-1) and the uncertain group 0. By setting different signs and even different weights, we expect to reinforce existing observed interactions and in the meanwhile, strengthen the negative impact of the highly negative interactions. Detailed comparison of the model with or without signed adjacency matrix in Table S1.

### Feedforward neural network classifier

Instead of directly using generative model to infer interactions, we take out embeddings ***z***_***i***_ contained in the matrix ***Z*** and further train a simple neural network as the final classifier. Correspondingly, the inputs to the classifier are concatenations of embeddings of two proteins, while the output label is a binary value representing whether there exist an interaction between the two proteins.

The performance of the classifier can be remarkably good without complex neural network structures since the embeddings already contain enough information and are highly representative in the learned low-dimensional vector space.

## Supplementary information

**Additional file 1** Detailed comparison of the model with or without signed adjacency matrix. Table S1. Detailed comparison of the model with or without signed adjacency matrix.

## Data Availability

The code and datasets during the current study are available in the GitHub repository: https://github.com/fangyangbit/S-VGAE.

## Abbreviations

PPIs: Protein-protein interactions
Y2H: Yeast two-hybrid screens
TAP: Tandem affinity purification
MS-PCI: Mass spectrometric protein complex identification
TAP-MS: Tandem affinity purification and mass spectrometry
Co-IP: Affinity chromatography and Co-Immunoprecipitation
KNN: k-Nearest neighbor
SVM: Support vector machine
SAE: Stacked autoencoder
DeepPPI: Deep neural networks for Protein Protein Interactions prediction
MDPN: Multimodal deep polynomial network
EnsDNN: Ensemble deep neural networks
LDA-ROF: Latent dirichlet allocation-random forest
ELM: Extreme learning machine
CS-SVM: Compressive sampling support vector machine
DFC: Deep fully connected network
DLSTM: Deep long short memory network
DIP: Database of interacting protein
C.elegans: Caenorhabditis elegans
E.coli: Escherichia coli
AC: Auto covariance
CT: Conjoint triad
LD: Local descriptor
GCN: Graph convolutional network
ReLU: Rectified linear unit
LDA-SVM: Latent dirichlet allocation-support vector machine
VGAE: Variational graph auto-encoder
S-VGAE: Signed variational graph auto-encoder
HPRD: Human protein reference database
SMILES: Simplified molecular input line entry specification
DDIs: Drug-drug interactions
