# Supplementary material for: Graph-based prediction of Protein-protein interactions with attributed signed graph embedding
Source: BMC Bioinformatics. 2020 Jul 21;21:323. doi: 10.1186/s12859-020-03646-8 (PMC7372763; doi:10.1186/s12859-020-03646-8)
Supplement: Supplementary file 1 — Additional file 1 Detailed comparison of the model with or without signed adjacency matrix. Table S1. Detailed comparison of the model with or without signed adjacency matrix. [file 12859_2020_3646_MOESM1_ESM.docx]

**Additional File 1-** **Detailed comparison of the model with or without signed adjacency matrix**

**Table S1.** Detailed comparison of the model with or without signed adjacency matrix

| Dataset | F1(%)/A | F1(%)/B |
| --- | --- | --- |
| HPRD | 99.15 ± 0.12 | 98.89 ± 0.03 |
| Human | 98.78 ± 0.24 | 98.43 ± 0.21 |
| E.coli | 98.92 ± 0.54 | 98.90 ± 0.40 |
| Drosophila | 99.80 ± 0.15 | 99.75 ± 0.22 |
| C.elegan | 99.25 ± 0.33 | 99.12 ± 0.16 |

A: the model is with signed adjacency matrix.

B: the model is with common adjacency matrix.
